# Regulatory T Cells Across Oral Diseases: A Narrative Review on a Functional Perspective

**DOI:** 10.3390/ijms27198555

**Published:** 2026-09-24

**Authors:** Yu Katsuma, Eiko Sakai, Yoshinori Sumita

**Affiliations:** Department of Medical Research and Development for Oral Disease, Nagasaki University Graduate School of Biomedical Sciences, Nagasaki 852-8588, Japan; bb55324203@ms.nagasaki-u.ac.jp (Y.K.); y-sumita@nagasaki-u.ac.jp (Y.S.)

**Keywords:** regulatory T cells, FOXP3, immunosuppressive function, Th17/Treg balance, oral disease, Sjögren’s disease, oral lichen planus, periodontitis, peri-implantitis, oral squamous cell carcinoma, medication-related osteonecrosis of the jaw

## Abstract

Regulatory T cells (Tregs) maintain peripheral immune tolerance and are involved in various oral diseases with markedly different characteristics. In the oral field, Tregs have largely been discussed through reports on their distribution and numbers, and interpretations of whether their role is protective or pathogenic. Current identification markers do not directly assess the suppressive function, and reports on this function remain limited. This review focuses on the suppressive function of Tregs in the organization of oral diseases. Cells may express FOXP3 without exerting stable suppressive function, and the suppressive capacity of the same FOXP3^+^ cells may vary with TSDR status and the inflammatory environment. In Sjögren’s disease, oral lichen planus, periodontitis, and peri-implantitis, inflammation does not resolve even when Treg numbers are increased or maintained. In contrast, Treg suppressive function is enhanced in oral squamous cell carcinoma. Medication-related osteonecrosis of the jaw differs in that a reduction in Tregs induced by the causative drugs is itself what governs the condition. Attending to the functional state allows these conditions to be organized coherently and supports a therapeutic conception of Treg control that differs in direction according to the disease: weakening the function where it is enhanced, and restoring it where it is impaired.

## 1. Introduction

Regulatory T cells (Tregs) are cells that suppress immune responses to maintain self-tolerance and tissue homeostasis [1]; their excess, deficiency, or functional abnormalities are implicated in various diseases. The oral cavity serves as the entrance to the gastrointestinal and respiratory tracts and features a unique structure between hard tissues and mucosa. It is constantly exposed to physical stimuli, resident microbiota, and exogenous antigens [2], and is rich in immune cells [3,4]. Therefore, the constant maintenance of immune homeostasis is required, and Tregs play a significant role in this process. The role of Tregs in multiple oral diseases with vastly different pathological characteristics has been examined from various perspectives, and their distribution, protective or pathogenic roles, and regulatory mechanisms have been characterized for each disease [5]. However, it is difficult to consistently understand their behavior from their distribution and reported roles across various diseases. Several diseases have been reported in which Treg levels increase despite the progression of inflammation and tissue destruction [6,7].

To account for this, it is necessary to examine whether Tregs actually exert suppression. Identification using current markers does not directly assess the actual suppressive function of these cells, and forkhead box P3 (FOXP3) expression alone does not guarantee stable suppressive capacity. This perspective leads to the reevaluation of disease-specific treatment strategies aimed at normalizing immune tolerance. Simultaneously, this may provide clues for identifying factors to target to regulate Treg function.

In this review, we examine six conditions—Sjögren’s disease, oral lichen planus, periodontitis, peri-implantitis, oral squamous cell carcinoma, and medication-related osteonecrosis of the jaw—from the perspective of the functional status of Tregs. First, we provide an overview of Treg differentiation, suppression mechanisms, stability, and plasticity, and summarize the molecular basis underlying the mismatch between identification markers and their suppressive functions. We then review the current findings for each disease, discuss the interpretation and significance of research results, and explore current trends and developments in research. By integrating these perspectives, we explore future research directions and discuss emerging research questions.

## 2. Tregs Biology

### 2.1. Introduction to Tregs and FOXP3

Tregs are a subset of CD4^+^ T cells responsible for maintaining peripheral immune tolerance and homeostasis [8]. The master regulatory factor governing cell differentiation and function is the transcription factor FOXP3 [9,10]. FOXP3 regulates the expression of numerous target genes in the nucleus [11]; while activating molecules such as CD25 (the IL-2 receptor α chain) and CTLA-4 (cytotoxic T-lymphocyte-associated antigen 4), which characterize the Treg phenotype and suppressive function, it simultaneously suppresses programs associated with effector T cells, such as IL-2 production [12]. Humans with FOXP3 deficiency develop immune dysregulation, polyendocrinopathy, enteropathy, X-linked (IPEX) syndrome, and scurfy mice develop lethal systemic autoimmunity, demonstrating that Tregs play a central role in self-tolerance [13,14].

Based on their origin, Tregs are broadly classified into thymus-derived Tregs (tTregs), which differentiate in the thymus, and peripherally induced Tregs (pTregs), which are induced from conventional CD4^+^ T cells in the periphery in the presence of transforming growth factor β (TGF-β) [15,16]. Furthermore, cells induced in vitro in the presence of TGF-β are called inducible Tregs (iTregs); however, they are not considered to be the same population as pTregs, and their phenotype is relatively unstable [15,16]. Phenotypically, Tregs exhibit high expression of CD25 and low expression of CD127 (IL-7 receptor α chain) [17,18]. While these are representative identification markers, the indicators used in the studies reviewed here are not uniform. They vary depending on the method used, including FOXP3 immunostaining, flow cytometry for CD4^+^CD25^+^ cells or for those with high CD25 and low CD127 expression, and mRNA analysis of transcription factors. Because none of these markers directly measure suppressive function, the identified cell populations do not match when different markers are used. These differences must be considered when comparing Tregs between different diseases and studies.

### 2.2. Suppressive Mechanisms

There are several mechanisms by which Tregs suppress immune responses, which can be broadly categorized into four types [19]. The first mechanism is mediated by suppressive cytokines. IL-10, TGF-β, and IL-35 suppress effector T cell activation and inhibit local inflammation [20,21,22]. The second mechanism is cytotoxicity-mediated; some activated Tregs induce apoptosis in NK cells and CD8^+^ T cells in a perforin- and granzyme-dependent manner. A reduction in the number of these effector cells suppresses cytotoxic responses [23]. The third mechanism involves metabolic interference. Tregs highly express CD25 and consume surrounding IL-2, thereby inducing apoptosis in effector T cells that are deprived of survival signals [24]. In addition, cell surface ectonucleotidases CD39 and CD73 convert extracellular nucleotides into adenosine, which suppresses effector T cells via the A2A adenosine receptor [25]. The fourth mechanism is the functional suppression of antigen-presenting cells. CTLA-4 removes the costimulatory molecules CD80/CD86 from dendritic cells and other antigen-presenting cells via transendocytosis, thereby depriving T cells of the costimulation necessary for their activation [26]. Furthermore, unlike effector T cells, which rely heavily on glycolysis, Tregs depend on a unique metabolic program centered on fatty acid oxidation [27]. These metabolic features contribute to Treg function and stability. Lipid metabolism in intratumoral Tregs supports their immunosuppressive function [28,29].

### 2.3. FOXP3 Stability and TSDR

Epigenetic regulation of the FOXP3 locus, particularly demethylation of the Treg-specific demethylated region (TSDR, also known as conserved noncoding sequence 2 (CNS2)), is key to the stable expression of FOXP3 and maintenance of its regulatory function [30]. The FOXP3 locus contains multiple conserved noncoding sequences (CNSs) with specific functions. CNS1, which contains a TGF-β–NFAT response element, is involved in the induction of pTregs in the periphery, whereas through CpG demethylation and Cbf-β–Runx1-dependent binding of FOXP3 itself, CNS2 (TSDR) functions as an element of “cellular memory” that stably maintains FOXP3 expression even in daughter cells after division [31]. In thymus-derived Tregs (tTregs), the TSDR is completely demethylated, ensuring stable FOXP3 expression. In contrast, in iTregs induced in vitro in the presence of TGF-β, the TSDR remains largely methylated, and FOXP3 expression is unstable and prone to loss [32]. However, it has been reported that in vivo-induced pTregs have a demethylated TSDR and exhibit stable FOXP3 expression; therefore, they cannot be treated as equivalent to iTregs [16]. Although conventional human T cells transiently express FOXP3 upon activation, these cells do not possess suppressive functions [33]. Thus, FOXP3 expression alone does not guarantee Treg function. Furthermore, when TSDR demethylation was specifically targeted in human non-Treg CD4^+^ T cells, FOXP3 expression was induced at physiological levels and with a physiological isoform distribution, yet this was accompanied by neither upregulation of Treg markers nor acquisition of suppressive capacity [34]. Demethylation of the TSDR is required for stable FOXP3 expression, but is not sufficient to give rise to functional Tregs [34]. Moreover, even when the TSDR is demethylated at sites of chronic inflammation, stable FOXP3 expression is not maintained [35]. Whether this loss of stability is accompanied by changes in the TSDR varies depending on the disease.

### 2.4. Plasticity and the Th17–Treg Axis

Tregs and inflammatory T helper 17 (Th17) cells are closely related in their differentiation. Although both differentiate from naive CD4^+^ T cells, their fate is determined by the cytokine environment. In the presence of TGF-β alone, FOXP3 is induced, leading to a shift toward Tregs; however, when IL-6 is added to TGF-β, retinoic acid receptor-related orphan receptor γt (RORγt) is induced [36], and differentiation into Th17 cells is prioritized [37]. Therefore, Th17 cells and Tregs are often viewed as being in an antagonistic equilibrium (Th17/Treg balance), and shifts in this balance are considered to influence the progression of inflammation and the maintenance of tissue homeostasis. These two cell types differ in their metabolic activities. While Th17 cells depend on de novo fatty acid synthesis mediated by acetyl-CoA carboxylase 1 (ACC1), Tregs take up and utilize exogenous fatty acids; therefore, inhibition of fatty acid synthesis suppresses Th17 cells and favors Tregs [38]. Furthermore, Tregs expressing FOXP3 are not necessarily immutable cells that have completed their differentiation. Under inflammatory conditions, particularly in the presence of IL-6, FOXP3 expression becomes unstable, and FOXP3^+^ cells may lose their suppressive properties and adopt Th17-like characteristics, such as IL-17 production [39]. Cells that lose FOXP3 expression in this manner are called exFoxp3 cells and can produce inflammatory cytokines that may promote disease progression [40,41]. The Th17/Treg balance and plasticity are recurring themes across the diseases discussed in this review. In periodontitis, FOXP3^+^ cells exhibit the plasticity to produce IL-17 [7], and in oral lichen planus, an increase in the IL-17-producing fraction of FOXP3^+^ cells in peripheral blood has also been reported [6]. In medication-related osteonecrosis of the jaw (MRONJ), the causative drug shifts this balance toward Th17 cells, which serve as the starting point of the disease process [42].

### 2.5. Identification and Suppressive Function of Tregs

The current markers used to identify Tregs do not directly assess whether these cells actually exert suppression. FOXP3^+^ cells are relatively easy to detect via flow cytometry or tissue staining, and many studies have used them as indicators. However, depending on the state of the TSDR and the inflammatory environment, the immunosuppressive capacity of even the same FOXP3^+^ cells can vary significantly; owing to plasticity, they may shift toward promoting inflammation, or their immunosuppressive activity may be enhanced through metabolic adaptation. The characteristics of the reported Treg populations vary depending on the markers used. In the following sections, the central question is how the suppressive function has been examined, and the findings reported for Tregs in each disease are reconsidered on that basis. Studies that directly evaluate suppressive function are limited, and this deficiency is treated in this review as a cross-disease challenge. As outlined above, Treg suppressive function is not fixed but is continuously shaped by the epigenetic, metabolic, and microenvironmental pathways that regulate FOXP3 stability and Th17/Treg plasticity. These pathways governing the functional stability of Tregs are summarized in Figure 1.

## 3. Treg and Oral Diseases

### 3.1. Sjögren’s Disease

Sjögren’s disease (SjD) is an autoimmune disease that primarily affects the salivary and lacrimal glands. It is characterized by lymphocytic infiltration of the exocrine glands and the production of autoantibodies, including anti-SS-A/Ro antibodies. It may spread systemically, accompanied by lymphocytic infiltration of various organs and hypergammaglobulinemia [43]. Although autoreactive B and T cells are involved, the pathophysiology of this disease remains incompletely elucidated [44]. SjD also frequently occurs together with other autoimmune diseases. In a cross-sectional series of 410 patients, 32.6% had at least one additional autoimmune disease, most often autoimmune thyroid disease, rheumatoid arthritis, or systemic lupus erythematosus [45], and a nationwide cohort study found that patients with SjD subsequently developed systemic lupus erythematosus or rheumatoid arthritis far more often than matched controls [46]. That tolerance fails against tissues of several different origins in the same patient suggests that the defect is not confined to the exocrine glands but lies in the mechanisms maintaining systemic self-tolerance, in which Tregs occupy a central position.

The role of Tregs in SjD remains unclear, and the debate continues. Regarding Treg counts, reports on the frequency of Tregs in the blood of patients with SjD are divided as follows: some indicate an increase [47], others a decrease [48], and others no change [49]; thus, no consistent trend is evident. This inconsistency may be attributable not only to differences among patient cohorts but also to the use of different markers to measure Tregs, which makes comparisons across studies difficult [50]. Regarding the number of Tregs infiltrating tissues, some reports indicate a marked decrease in the salivary glands [48], whereas others report an increase that correlates positively with the degree of infiltration [49]; therefore, the association between Treg infiltration and disease status and severity remains unclear [51]. Furthermore, although increasing the number of Tregs can promote immune tolerance, safety concerns have been raised regarding whether this might increase the risk of malignancy, particularly non-Hodgkin’s lymphoma, in patients with SjD [52]. It is difficult to assess the state of Tregs or their benefit from counts alone, and it is necessary to directly examine whether Tregs are, in fact, exerting suppression.

In recent years, important findings have been reported regarding this issue. Although it had already been demonstrated that Treg function and differentiation depend on STIM1/2-mediated store-operated Ca^2+^ entry (SOCE) [53], it was subsequently revealed that mice with a Foxp3^+^ Treg-specific deficiency in STIM1/2 develop a phenotype that meets all classification criteria for human SjD [44]. In these mutant mice, Tregs in the cervical lymph nodes increased in both absolute numbers and frequencies but could not prevent disease onset. CD4^+^CD25^−^ T cells (non-Tregs) were isolated from the cervical lymph nodes of *Stim1/2^Foxp3^* mice and injected into lymphocyte-deficient *Rag1*^−/−^ mice; this transfer alone was sufficient to induce a Sjögren’s-like disease. Effector CD4^+^ T cells that acquire autoreactivity in the presence of dysfunctional Tregs are sufficient on their own to trigger the disease. Furthermore, when these autoreactive effector CD4^+^ T cells were co-injected with equal numbers of normal (wild-type [WT]) Tregs or dysfunctional (Stim1/2-deficient) Tregs, the WT Tregs suppressed lymphocyte infiltration into the lacrimal gland and IFN-γ production, whereas dysfunctional Tregs were unable to suppress these processes despite being present in equal numbers. However, although no significant improvement was observed in saliva production, there was a significant improvement in tear production [44]. This co-injection experiment, in which equal numbers of Tregs were compared, directly demonstrated that the success or failure of suppression depends on the functional state [44]. These autoreactive effector CD4^+^ T cells are Th1 cells that significantly overexpress T-bet and moderately downregulate GATA3 expression. In experiments to knock down *Ifng* in these cells, salivary and lacrimal secretions were restored, demonstrating that IFN-γ is a key factor in glandular dysfunction. IFN-γ did not affect immune cell infiltration; in human salivary gland cells, it reduced the expression of the M3 receptor (CHRM3) and the P2X7 receptor, thereby inhibiting calcium influx and reducing saliva secretion [44].

Although these findings are based on cellular and mouse-level studies, single-cell RNA sequencing of peripheral blood mononuclear cells from human patients with SjD also revealed that in CD4^+^ T cells, gene expression programs associated with Th1 and interferon signaling were upregulated, whereas programs associated with memory Treg function were significantly downregulated [44]. However, the frequencies of both naive and memory FOXP3^+^ Tregs in the blood were equivalent in patients and controls [44]. These findings suggest that, as in mice, Treg dysfunction and an enhanced Th1/IFN-γ response are closely involved in the pathogenesis of SjD in humans. Other autoreactive lymphocytes, in addition to Th1 cells, are involved in pathological conditions caused by dysfunctional Tregs. In patients’ salivary glands, Ro60-specific CD4^+^ T cells were concentrated in the T follicular helper/T peripheral helper (Tfh/Tph) cluster rather than in the Treg cluster, indicating a breakdown in immune tolerance [54]. Tfh/Tph cells collaborate with B cells to produce autoantibodies. The relationship between these autoreactive lymphocytes and the breakdown of tolerance associated with Treg dysfunction remains unclear and requires further investigation.

Therapeutic interventions have also been attempted for IFN-γ-driven pathologies. In mouse experiments, the administration of baricitinib (a JAK1/2 inhibitor) suppressed IFN signaling by inhibiting the JAK/STAT pathway, resulting in improvements in both infiltration and secretion in the salivary and lacrimal glands [44]. This approach is novel in that it targets the “upstream pathophysiological mechanism” (the IFN-γ pathway originating from Treg dysfunction), rather than broadly suppressing inflammation or providing symptomatic treatment, which have traditionally been the mainstay of SjD management [43]. Furthermore, approaches aimed at restoring Treg function (such as low-dose IL-2) are also being investigated, and improvements in salivary secretion, first observed in mouse models [55], have since been reported in randomized clinical trials involving patients [56]. These findings demonstrate the potential of pathogenesis-specific immunotherapy, shifting from the suppression of inflammation and immune responses to the restoration of immune tolerance.

Many of the findings presented here are based on mouse models. In humans, Treg function itself has not been directly demonstrated and remains limited to indirect inferences based on gene signatures. In the single-cell analysis described above, genes involved in Ca^2+^ homeostasis could not be analyzed in Tregs because of insufficient coverage, and in effector CD4^+^ T cells *ORAI1*, *ATP2B4*, and *ITPR1* were moderately increased; whether these changes relate to alterations in Treg function remains to be determined [44]. Furthermore, in human studies, reports indicated that the suppressive function of patient-derived CD4^+^CD25^+^ T cells [48] and CD4^+^CD25^high^ cells [47] did not differ from that of controls. However, because these reports pertain to different cell subsets, a direct comparison is difficult. In addition, when STAT5 activation in response to IL-2 stimulation was used as an indicator, Tregs exhibited reduced responsiveness, whereas their frequency remained unchanged [57]. In other words, conclusions vary depending on the indicators used to evaluate the suppressive function. Therefore, further verification of the actual state of Treg dysfunction in human SjD and its therapeutic applications is necessary.

### 3.2. Oral Lichen Planus (OLP)

Oral lichen planus (OLP) is a chronic T-cell-mediated inflammatory disease that primarily affects the oral mucosa. It is characterized by damage to the basal layer of the epithelium and lymphocytic infiltration into the subepithelial layer. A persistent T-cell immune response against epithelial cells drives the disease process [58,59]; however, the underlying pathogenesis remains poorly understood. The role of Tregs in OLP has been reported primarily by quantifying markers. Several reports have shown an increase in FOXP3^+^ Tregs in affected tissues and peripheral blood [60,61,62], suggesting that Tregs are involved in pathogenesis. These reports also indicate that FOXP3^+^ cells are predominantly of the low-activity reticular type and are less common in cases with an erosive type or longer duration of disease [60,61]. However, most of these studies were limited to measuring quantitative indicators, such as cell counts and marker molecule expression, and very few studies have evaluated whether these cells exert an inhibitory effect. In OLP, one study directly examined the suppressive function of the increased FOXP3^+^ cells in humans [6].

According to this report, Tregs increased in patients with OLP, as indicated by multiple markers. In peripheral blood, the proportion of CD4^+^CD25^+^CD127^low/−^ cells—which are widely used to identify Tregs—was significantly higher in both the reticular and erosive subtypes compared with the control group [6]. In the lesion tissue, while FOXP3^+^ cells were rarely observed in the normal control mucosa, there was increased infiltration of FOXP3^+^ cells into the lamina propria of the OLP lesion [6]. Furthermore, this increase was not limited to the cell number; the amount of FOXP3 protein expressed per Treg was higher than in the control group [6], and, consistent with this, FOXP3 mRNA expression was also elevated [6]. In OLP, both an increase in the number of Tregs and elevated FOXP3 expression were observed, indicating that the suppressive side of the immune response was quantitatively enhanced.

Nevertheless, the suppressive function of Tregs is impaired. In an in vitro suppression assay in which CD4^+^CD25^+^ T cells (Tregs) derived from patients with OLP were co-cultured with their own CD4^+^CD25^−^ conventional T cells, the Tregs from patients with OLP showed a significantly lower ability to suppress the proliferation of conventional T cells compared with the control group [6]. Abnormalities in the suppressive function have also been reported at lesion sites. In OLP lesional tissue, only a few cells co-express FOXP3 and sialyl-Lewis X (CD15s), a marker of effector Tregs that possess suppressive capacity [63]. The proportion of FOXP3^+^CD15s^+^ cells is significantly higher in the reticular form than in the normal oral mucosa and other forms of OLP [63]. Findings consistent with these functional impairments have been reported previously. The expression of TGF-β—a key cytokine responsible for Treg suppressive function—was reduced in patients at both the mRNA and serum levels [6]. Furthermore, the proportion of CD4^+^FOXP3^+^ cells producing the inflammatory cytokine IL-17 was significantly increased in patients [6]. This suggests that the phenotype of FOXP3^+^ cells, which normally play a suppressive role, shifts toward promoting inflammation, particularly IL-17 production. In contrast, no significant difference was observed in the proportion of IFN-γ-producing FOXP3^+^ cells [6]. All the above findings were based on the analyses of peripheral blood samples. In lesion tissue, IL-17 production was not detected in FOXP3^+^ cells, and IL-17-positive cells have been reported to be CD3-negative and not T cells [63]. Furthermore, some FOXP3^+^ cells express T-bet [63], and it is unclear whether the direction of plasticity is consistent between Tregs in the peripheral blood and those at the lesion site.

Moreover, these reductions in TGF-β and increases in FOXP3^+^IL-17^+^ cells were observed in conditions associated with dysfunction; however, the mechanisms underlying this dysfunction have not been demonstrated. Molecular mechanisms, such as destabilization of FOXP3 or underlying epigenetic regulatory abnormalities, which explain why Tregs lose their suppressive capacity, were not explored in depth in this study. It has been reported, however, that being FOXP3^+^ does not in itself guarantee suppressive function [33], that demethylation of the TSDR alone does not yield a functional Treg [34], and that at sites of chronic inflammation stable FOXP3 expression is not maintained even when the TSDR is demethylated [35]. The state observed in OLP, in which a quantitative increase in Tregs was not accompanied by an immunosuppressive function, is consistent with the concept of FOXP3 instability at sites of inflammation. However, no studies have directly examined FOXP3 instability or TSDR demethylation in Tregs within OLP lesions. One factor complicating this verification is that TSDR methylation analysis is not suitable for single-cell-level assessment of tissues and cannot identify cellular localization [63].

These findings indicate that Treg dysfunction in OLP occurs at the level of suppressive function. Therefore, the therapeutic challenge lies in restoring lost suppressive function or supplementing it with functional Tregs. In fact, it has been shown that deficiency in Foxp3 or IL-2 in mice causes oral mucosal inflammation, which can be suppressed by simultaneously introducing functional Tregs [64]. However, that study was based on a mouse infusion model and did not provide OLP-specific evidence. Furthermore, exogenous supplementation with functional Tregs is distinct from restoring dysfunctional Tregs. A previous review identified OLP, a precancerous lesion, as a target for the removal or suppression of FOXP3^+^ Tregs [5]. Although OLP is classified as an oral precancerous lesion [65], the rate of malignant transformation under strict diagnostic criteria has been reported to be 0.44% [66]. Therefore, caution should be exercised when considering Tregs as targets for elimination based solely on the suppression of malignant transformation. Treatments that directly target functional recovery in OLP have not yet been developed.

The greatest limitation in interpreting this section is that direct evidence of impaired Treg function in OLP comes from suppression assays conducted in a small number of cases (three reticular-type cases and three control cases) [6], and no functional assays have been performed for the erosive type. Because the key data supporting the claim of functional impairment are based on a small number of cases, this finding needs to be confirmed through large-scale functional validation studies.

### 3.3. Periodontitis

Periodontitis is a chronic, multifactorial inflammatory disease associated with plaque biofilm formation accompanied by dysbiosis (an aberrant microbial community) [67,68]. It is characterized by the progressive destruction of periodontal tissues, manifesting as attachment loss and alveolar bone resorption [69]. This alveolar bone resorption is mediated by osteoclasts [70] and is governed by the balance between Th17 cells and Tregs [7,71,72].

Tregs are protective cells that support periodontal tissue homeostasis by secreting anti-inflammatory cytokines (IL-10, TGF-β) and inhibiting osteoclast formation; they suppress osteoclast differentiation in co-culture with bone marrow macrophages [7]. In vivo inhibition of Treg function by an anti-GITR antibody in murine experimental periodontitis aggravates alveolar bone resorption and inflammatory cell infiltration [73]. In human lesions, FOXP3^+^ and CD25^+^ cells are increased relative to healthy controls, although they are reported to be fewer in stage IV, grade C than in stage III, grade B periodontitis [74]. However, as described below, an increase in these cells does not necessarily mean that they are the cells carrying out suppression. In experimental periodontitis in mice, Tregs sorted from draining lymph nodes have been reported to exhibit reduced Foxp3 expression and diminished capacity to inhibit osteoclast formation [7]. Therefore, the therapeutic challenge in this disease lies in securing functionally intact Tregs at the lesion site, which requires both their recruitment to the site and the restoration of the suppressive function lost under inflammation.

Alvarez et al. examined this pathology in a ligature-induced murine model of periodontitis. As periodontitis progressed, mediators associated with both Th17 cells and Tregs (IL-6, IL-17A, IL-17F, RANKL, IL-10, TGF-β, and GITR) increased in periodontal lesions [7]. In cervical lymph nodes, the numbers of Th17 cells and Tregs also increased with the progression of periodontitis [7]. Expanded Th17 cells promote RANKL expression via IL-17, thereby driving alveolar bone resorption through osteoclast formation and activation [7,72,75]. In both human and murine periodontitis, the dysbiosis-driven Th17 response has been shown to contribute to tissue destruction [76]. In contrast, Tregs protect the bones by suppressing osteoclast formation. The maintenance or loss of alveolar bone depends on the balance between Th17-mediated bone resorption and Treg-mediated protection. However, the progression of periodontitis despite an increase in FOXP3^+^ cells indicates that cell number alone does not necessarily reflect the suppressive capacity of this population.

Tregs in periodontitis have been shown to be phenotypically destabilized. In CD4^+^CD25^+^Foxp3^+^ cells sorted from the cervical lymph nodes of mice with periodontitis, Foxp3 expression (mean fluorescence intensity) was reduced by approximately 28% compared with before induction, while both the proportion and the absolute number of IL-17A-producing Tregs were significantly increased [7]. Likewise, gene expression analysis showed that Tregs derived from patients with periodontitis had elevated expression of Th17-associated factors, yielding an expression profile distinct from that of healthy individuals [7]. However, this represents a state in which cells sorted as CD4^+^CD25^+^Foxp3^+^ acquire Th17 characteristics. Since exFoxp3 cells lost Foxp3 expression, they were excluded during the sorting step; this finding cannot be interpreted as a conversion of Tregs into exFoxp3 cells. The inflammatory milieu underlies this phenomenon. IL-6 expression is high in the gingiva in periodontitis [7], and, as described in Section 2, sustained IL-6 in the presence of TGF-β destabilizes Foxp3 in Tregs and promotes the acquisition of IL-17-producing capacity [39].

This destabilization extends to their function. When Tregs from mice with periodontitis were co-cultured with bone marrow macrophages in the presence of RANKL and M-CSF, significantly more TRAP-positive osteoclasts were formed than those from healthy controls, corresponding to a reduction of more than 30% in the suppressive capacity of Tregs [7]. TRAP-positive cells were also significantly larger [7]. That is, increased Tregs have reduced anti-osteoclastogenic capacity and are functionally impaired.

However, Tregs must reach the site of inflammation to protect bone. CCL22, a chemokine produced locally in periodontal lesions, recruits circulating CCR4^+^ Tregs to the lesions, thereby suppressing bone resorption [77]. In mice lacking CCR4, Treg migration is markedly impaired, and both bone resorption and pro-inflammatory and osteoclastogenic cytokine levels are increased [77]. Adoptive transfer of CCR4^+^ Tregs into these mice restored the exacerbated phenotype to levels comparable to those in wild-type mice [77]. However, it should be noted that the functional impairment shown by Alvarez et al. [7] was in Tregs of the draining lymph nodes, the site where they are generated, whereas the cells that actually protect the bone are the Tregs recruited to the lesion. Whether the functional impairment observed in the lymph nodes also applies to Tregs in the lesion remains to be determined; the two should be examined separately. Recruitment to the lesion and maintenance of the suppressive function are therapeutic targets. Thus, the protection of the alveolar bone in periodontitis depends on whether functionally competent Tregs can be recruited to the lesion in sufficient numbers.

Efforts to recruit Tregs to local sites are being pursued, and sustained release of CCL22 has been shown to recruit Tregs and reduce alveolar bone resorption in both mice and dogs [78]. Functionally, approaches are being explored to restore and maintain Foxp3 stability and suppressive function, which are compromised in an inflammatory environment. IL-2 supplementation, enhancement of Treg differentiation and suppression of Th17 differentiation by all-trans retinoic acid (ATRA), IL-33 administration, adoptive transfer of functional Tregs, and IL-17 inhibition to restrain Th17 cells have been proposed as candidates [72]. However, because these approaches target Tregs and the immune response, they require careful balancing of the risks of systemic immunosuppression [72].

The principal limitation of the findings presented in this section is that the molecular basis of Foxp3 destabilization at the center of this functional impairment remains unknown. Methylation of the TSDR was examined but showed no significant change and did not account for the reduction in Foxp3 [7]. Elucidating the molecular mechanisms driving this destabilization is a task for future studies.

Periodontitis is a condition in which Tregs increase in number while Foxp3 stability and anti-osteoclastogenic capacity decline. Both the increase in enumerated cells and the decline in suppressive function have been demonstrated in a single experimental system, and it is clear that enumeration with current markers does not reflect the state of Tregs.

### 3.4. Peri-Implantitis

Peri-implantitis is a microbially driven inflammatory disease caused by dysbiosis of the oral microbiota and is characterized by inflammation of the peri-implant mucosa and progressive resorption of the supporting bone [79]. It shares many pathological features with periodontitis, but the area of its inflammatory infiltrate is more than twice that of periodontitis, with significantly greater numbers of plasma cells, macrophages, and polymorphonuclear leukocytes [80], and its progression is considered faster than that of periodontitis [79]. The absence of a periodontal ligament and poor vascularity, together with foreign-body stimuli such as titanium particles, may contribute to these differences. In the search for diagnostic biomarkers using peri-implant crevicular fluid (PICF), levels of sRANKL, which promotes osteoclastogenesis, its antagonistic decoy receptor osteoprotegerin (OPG), and sclerostin, an osteocyte-derived Wnt inhibitor that suppresses bone formation, have been reported to be significantly higher in patients with peri-implantitis than in healthy controls [81].

As described below, in peri-implantitis as well as in periodontitis, both FOXP3 expression and the number of FOXP3^+^ cells are increased in the lesion. However, the stability and composition of the molecules involved in the suppression differ from those in healthy individuals, and whether the suppressive function is preserved has not been directly confirmed.

Th17-related cytokines have been detected in peri-implantitis lesions. Mardegan et al. compared biopsies of healthy peri-implant tissue and peri-implantitis tissue (20 each) using RT-PCR and showed that IL-23 mRNA was higher and TGF-β lower in peri-implantitis, whereas IL-17 mRNA did not differ between the two groups [82]. IL-23 expression correlated positively with radiographic bone loss and negatively with TGF-β [82]. The authors interpreted these findings as a shift towards Th17 predominance, but these were findings at the tissue mRNA level, and IL-17 itself was not increased [82]. Gonçalves et al. compared PICF before therapy and three months after in 20 patients with peri-implant mucositis (non-surgical therapy) and 17 patients with peri-implantitis (surgical therapy), and showed significant reductions in IL-17A and TNF-α in the mucositis group and in IL-1β, IL-6, and TNF-α in the peri-implantitis group [83]. The reduction in IL-17A levels in the peri-implantitis group was not significant [83]. IL-17 does not generate osteoclasts on its own; in the presence of RANKL, it promotes osteoclast formation, thereby advancing bone resorption [84]. A link between reduced IL-17A levels and reduced bone destruction has not been demonstrated in human peri-implantitis; however, Th17-related cytokines have been shown to vary in ways that may correspond to the degree of peri-implant inflammation.

Changes in the phenotype of Tregs have been reported. Giro et al. collected soft tissue biopsies from 15 healthy peri-implant tissues and 20 peri-implantitis lesions and compared transcription factor mRNA expression levels cross-sectionally using RT-qPCR [85]. Both RORγt, which defines Th17 cells [36], and FOXP3, which defines Tregs, were significantly increased in the lesions [85]. This measurement captures mRNA from the whole tissue rather than individual cells; however, based on the simultaneous increase in both transcription factors, Giro et al. raised the possibility of an increase in hybrid Th17–Treg cells co-expressing FOXP3 and RORγt within the same cell [85]. This was a cross-sectional, preliminary report; progression over time was not assessed, and no analyses at the cellular or protein levels were performed [85]. Giro et al. [85] went no further than stating that these transcription factor levels could serve as predictive indicators of progression. In periodontitis, a subset of Foxp3^+^ cells in Foxp3 fate-tracked mice has been shown to transiently produce IL-17A during inflammation [86]. Tregs in peri-implantitis may acquire Th17 characteristics; however, no study has demonstrated this at the cellular level.

The composition of Treg-related molecules has been reported. Cafferata et al. compared granulation tissue obtained during surgical therapy for peri-implantitis (PI group, 23 cases) and granulation tissue from lesions that proceeded to explantation (PI-X group, 23 cases) with healthy peri-implant tissue obtained during second-stage surgery (H group, 20 cases) [87]. In both lesion types, FOXP3 gene expression was upregulated, and the number of FOXP3^+^ cells increased, whereas neuropilin-1 (NRP-1), which is related to Treg stability, was decreased in both mRNA and positively stained area, and the suppressive cytokine TGF-β1 was decreased at the protein level [87]. Suppressive cytokines were not uniformly decreased; IL-35 was increased at both the mRNA and protein levels, and IL-10 was unchanged [87]. NRP-1 is also not specific to Tregs; in healthy tissue, it is detected mainly in epithelial cells, and in the affected tissue, it is detected in macrophage-like cells, which limits the interpretation of this decrease as a change in Tregs [87]. The upregulation of FOXP3 and the decrease in NRP-1 correlated with clinical severity, including probing depth and vertical defect depth [87]. For FOXP3, however, mRNA expression in lesions that proceeded to explantation was lower than in surgically treated lesions, while the number of FOXP3^+^ cells did not differ between them; therefore, the picture depends on how the same molecule is measured [87].

The driver specific to peri-implantitis, and absent in periodontitis, is a foreign body composed of titanium particles. Kheder et al. compared gingival tissues from a total of 20 patients divided into three groups: failed implants (FI: peri-implantitis indicated for explantation), successful implants (SI), and no implants (NI), using whole-transcriptome analysis. They showed that gene sets related to M1/M2 macrophage polarization and lymphocyte proliferation were significantly enriched in FI compared with NI [88]. In vitro, M0 macrophages stimulated with TiO_2_ nanoparticles and microparticles (100 µg/mL, 24 h) significantly increased IL-1β, IL-8, and IL-18, whereas TNF-α remained unchanged [88]. Of these cytokines, blockade of IL-1β around titanium implants has been reported to prevent titanium particle-induced osteolysis [89]. With a foreign-body reaction superimposed on bacterial inflammation, bone destruction around implants that lack a periodontal ligament and are poorly vascularized is considered to advance.

Regarding treatment, as with periodontitis, the goal is to restore the stability of Tregs that have been altered by the inflammatory environment. Cafferata et al. stated that strategies aimed at restoring Treg function, stabilizing FOXP3 expression, and modulating NRP-1 and TGF-β1 signaling could serve as immunomodulatory approaches complementing conventional mechanical and antimicrobial therapy [87]. For example, they cited the targeted delivery of an NRP-1 agonist to promote both the restoration and maintenance of the immunoregulatory Treg phenotype and Treg-mediated resolution of inflammation by M2 macrophages [87]. The challenge specific to peri-implantitis is that control of the bacterial biofilm must be accompanied by measures to address the foreign-body stimulus of titanium particles. No intervention study targeting Tregs exists for this disease, and the inference rests on findings from periodontitis, together with preclinical proposals.

Therefore, peri-implantitis is a condition in which FOXP3^+^ cells increase, while the composition of the molecules involved in suppression is altered relative to health. Whether a hybrid Th17–Treg phenotype exists, whether Tregs in the lesion retain suppressive function, and how the conditions of a superimposed foreign-body reaction and a poorly vascularized environment lacking a periodontal ligament affect Treg dynamics remain tasks for future research.

### 3.5. Oral Squamous Cell Carcinoma (OSCC)

Oral squamous cell carcinoma (OSCC) is a malignant tumor arising from the epithelium of the oral mucosa and accounts for approximately 90% of oral malignancies [90]. Its progression involves the suppression of T cell-mediated antitumor immunity; Tregs are regarded as central mediators of this immune suppression [91]. In patients with OSCC, CD4^+^CD25^+^FOXP3^+^ cells expressing Treg markers are present in both peripheral blood and at the tumor site [92].

As with the diseases considered so far in this review, in OSCC the number of FOXP3^+^ cells does not reflect the strength of Treg function. The direction is the opposite of that in other diseases; in OSCC, the suppressive function of Tregs is excessive, restraining antitumor immunity and promoting tumor immune evasion. While the therapeutic approach for other diseases is to restore Treg function, the task in OSCC is to inhibit Treg function.

Gasparoto et al. showed that the frequency of CD4^+^CD25^+^ T cells in peripheral blood did not differ between patients with OSCC and healthy controls, but within this fraction, the proportions of cells positive for FOXP3, GITR, CD45RO, CD69, and IL-10 were higher in patients [92]. Sun et al. showed that while the overall Treg frequency in the peripheral blood of patients with OSCC did not differ from that of healthy donors (*p* = 0.269), effector Tregs with strong suppressive activity (CD45RA^−^FOXP3^high^) were increased (*p* = 0.006) and resting Tregs (CD45RA^+^FOXP3^low^) were decreased [93]. Purified peripheral blood Tregs from patients with OSCC suppress the proliferation of allogeneic T cells more strongly than peripheral blood Tregs from healthy controls [92]. Even when equal numbers of Tregs were compared, the suppressive function was stronger on the patient side. The abnormality of Tregs in OSCC lies in their enhanced function.

The tumor microenvironment of solid cancers features hypoxia, acidification, lactate accumulation, and nutrient depletion, and OSCC exhibits these properties as well. Metabolic reprogramming, through which Tregs adapt to this environment, is thought to support their suppressive functions [94]. In cancer, depletion of tryptophan by IDO (indoleamine 2,3-dioxygenase) and activation of the aryl hydrocarbon receptor (AHR) by its metabolite kynurenine promote Treg differentiation [95], and CD39/CD73, expressed by Tregs, generate adenosine, which suppresses effector T cells [96]. PI3K-Akt-mTOR regulates glucose uptake and glycolysis, and supports the survival and suppressive function of Tregs [94].

In OSCC, the corresponding findings are at the level of association: IDO expression is associated with poor prognosis; overexpression of CD73 in the tumor microenvironment is associated with poor prognosis; and spatial transcriptomic analysis has linked enhanced lactate utilization with Treg infiltration and high HIF1A expression [94].

These mechanisms have been demonstrated in mouse tumor models. In tumor-infiltrating Tregs, the fatty acid receptor CD36 is selectively upregulated and, through PPAR-β signaling, maintains mitochondrial fitness, allowing adaptation to a lactate-rich tumor microenvironment [28]. SREBP-mediated lipid synthesis is also required to maintain intratumoral Treg function [29]. Intratumoral Tregs use both glycolysis and fatty acid metabolism to proliferate, as confirmed in Tregs sorted from human liver cancer [97]. In OSCC, high CD36 expression has been reported to be associated with metastasis and the mitochondrial status of Tregs [94]. Furthermore, growth differentiation factor 15 (GDF15), produced in the tumor microenvironment, binds to CD48 on T cells and lowers the activity of ERK and AP-1, thereby reducing the expression of STUB1, an E3 ubiquitin ligase that directs FOXP3 for degradation [98]. FOXP3 is stabilized, and both the generation of induced Tregs and suppressive function of natural Tregs are enhanced. However, this was demonstrated in hepatocellular carcinoma, not in OSCC [98].

The mechanisms described so far operate within the Treg itself, but the behavior of Tregs in the tumor is also shaped by the cells around them. FAP^+^ fibroblasts have been reported to induce C1QC^+^ macrophages via WNT2, and these macrophages secrete CCL2 to drive Treg accumulation within the tumor [99]. Whether such stromal and myeloid interactions influence not only the number of Tregs but also their suppressive capacity remains to be clarified.

The relationship between the number of FOXP3^+^ cells infiltrating tumor tissue and prognosis is not uniform. In OSCC, a high number of FOXP3^+^ cells has been reported to be associated with poor prognosis [100], whereas in head and neck squamous cell carcinoma, there are also reports of an association with favorable prognosis [101,102]. This meta-analysis was limited to human papillomavirus (HPV)-negative cases [102]. HPV-positive head and neck squamous cell carcinoma is treated as a distinct disease entity with a different prognosis [102]. One reason proposed for this inconsistency is that FOXP3^+^ cells are not homogeneous, comprising both effector Tregs with suppressive capacity and FOXP3^+^ non-Treg cells that lack suppressive capacity and produce inflammatory cytokines [91]. In colorectal cancer, the relationship between FOXP3 expression levels and prognosis has been shown to be reversed depending on which of the two predominates [91].

Therapies targeting these pathways are currently being investigated. In head and neck squamous cell carcinoma, nivolumab, an antibody directed against programmed cell death protein 1 (PD-1) that reactivates T cells, has been shown to prolong overall survival compared with standard therapy [103]. Targeting the metabolic pathways of Tregs can be combined with immune checkpoint inhibitors (ICIs) to enhance their effects [94]. The combination of IDO inhibitors and ICIs is currently being evaluated in clinical trials, including for OSCC [94]. In mouse tumor models, the inhibition or deletion of the fatty acid receptor CD36 or lipid synthesis (SREBP) selectively reduces intratumoral Tregs and exerts antitumor effects in addition to anti-PD-1 therapy [28,29]. In hepatocellular carcinoma, a GDF15-neutralizing antibody has been reported to shrink tumors, reduce intratumoral Tregs, and enhance the effects of an anti-PD-1 antibody when co-administered [98]. However, none of these have been examined in OSCC.

Several reservations apply here. First, the relationship between the number of FOXP3^+^ cells and prognosis is not uniform, and it has been suggested that Tregs may suppress ineffective inflammation that promotes tumor progression [102]; restraining them uniformly could therefore work to the patient’s disadvantage. Second, for pathways shared by effector T cells, such as glycolysis and fatty acid oxidation, it is difficult to selectively target Tregs [94]. Third, apart from IDO inhibitors, most of these targets remain preclinical, and verification of their clinical efficacy in OSCC is a task for future studies.

### 3.6. Medication-Related Osteonecrosis of the Jaw (MRONJ)

Medication-related osteonecrosis of the jaw (MRONJ) is a refractory condition characterized by the progressive exposure and necrosis of the jaw in patients receiving bone resorption inhibitors (bisphosphonates and denosumab) or antiangiogenic agents. It is defined as the presence of exposed necrotic bone persisting for more than 8 weeks in the oral or maxillofacial region, or the ability to palpate bone through a fistula in the absence of a history of radiation therapy to the jaw or tumor metastasis [104]. Although it is often triggered by dental procedures such as tooth extraction, its pathogenesis remains poorly understood [42,105].

Previous studies have shown that immunological mechanisms are involved in the pathogenesis of MRONJ. In particular, an imbalance between Tregs and Th17 cells has been proposed to contribute to its onset. The causative drugs are thought to reduce Tregs and activate Th17 cells, leading to the breakdown of immune homeostasis, inflammation, and necrosis of the jawbone [42].

The immunological mechanisms of MRONJ have been primarily demonstrated in a mouse model of bisphosphonate-related osteonecrosis of the jaw (BRONJ). When tooth extraction is performed after administration of zoledronate and dexamethasone, lesions develop that replicate the key findings of human MRONJ, including non-healing open wounds, exposed necrotic bone, inflammatory cell infiltration, and bone sclerosis [42]. In this model, zoledronate administration significantly reduced the number of Tregs (CD4^+^CD25^+^Foxp3^+^) in the peripheral blood, while IL-17-producing Th17 cells were activated, thereby decreasing Treg/Th17 ratio [42]. Zoledronate drove this decrease in Tregs, whereas dexamethasone alone increased Tregs; however, their net number decreased when the two drugs were administered in combination [42]. Furthermore, in mice treated with zoledronate and dexamethasone, serum IL-10 levels decreased, weakening the anti-inflammatory response [42]. It is believed that these factors act in combination against the backdrop of long-term inflammation and impaired wound healing to cause jaw osteonecrosis.

However, the molecular mechanism by which zoledronate reduces Tregs remains unclear. One clue lies in the known pharmacological effects of nitrogen-containing bisphosphonates. Specifically, they inhibit farnesyl diphosphate (FPP) synthase in the mevalonate pathway [106]. As a result of this enzymatic reaction, the upstream metabolic intermediates IPP and DMAPP accumulate in monocytes and activate Vγ9Vδ2-type γδ T cells [107], whereas the downstream compounds FPP and geranylgeranyl diphosphate (GGPP) are depleted, leading to a decrease in protein prenylation. While the former may lead to inflammatory changes, because Vγ9Vδ2 γδ T cells and the phosphoantigen-sensing system that activates them are specific to humans and absent in mice, this pathway does not apply to mouse BRONJ-like models. On the other hand, experiments in which statins inhibited the mevalonate pathway (cholesterol and lipid metabolism, as well as prenylation) have shown that Treg proliferation and the acquisition of their suppressive function depend on this pathway [108]; since this mechanism holds true across species, it represents a more direct candidate for Treg depletion observed in mice. In other words, although the site of inhibition varies, it is conceivable that zoledronate could impair Treg proliferation and function by inhibiting this pathway. Kikuiri et al. demonstrated a decrease in Treg numbers, consistent with the finding that the mevalonate pathway promotes Treg proliferation [42].

Alternatively, a pathway mediated by changes in the cytokine environment has also been proposed. In a minipig model of BRONJ, bisphosphonate administration led to elevated serum IL-17 levels and a tendency toward decreased Treg numbers [109]. Furthermore, Li et al. argued that, because TGF-β alone promotes Treg differentiation while IL-6 promotes Th17 differentiation, the reduction in TGF-β and the increase in IL-6 caused by bisphosphonates could shift the balance toward Th17 cells and inhibit Treg differentiation [109]. However, both the intracellular mechanism mediated by the mevalonate pathway and the mechanism mediated by the cytokine environment remain as candidate explanations for the reduction in Tregs and have not been directly established in MRONJ. Elucidating the molecular mechanisms underlying the reduction in Tregs in MRONJ remains an issue for future research.

Kikuiri et al. [42] have demonstrated the therapeutic potential of this mechanism. When an anti-CD25 antibody was administered to mice treated with zoledronate and dexamethasone, the proportion of animals exhibiting exposed bone increased from 50% to 67%. Necrotic bone and inflammatory cell infiltration were observed in the extraction sockets, and bone healing was delayed [42]. In contrast, in the group that received systemic Treg infusion two days after tooth extraction, lesion formation was suppressed, and the mice followed a normal healing course characterized by epithelial coverage and bone formation [42]. In immunocompromised (beige nu/nu Xid) mice, osteoclast activity was suppressed in BRONJ-like lesions and was restored by Treg infusion; this observation was based on staining and was not quantified [42]. These findings indicate that osteoclast suppression, thought to result from the drug’s direct action, may also be influenced by the immune environment. In other words, Tregs play a protective role in the disease state, and restoring their numbers is a therapeutic target. A similar therapeutic effect was observed following the systemic administration of mesenchymal stem cells (MSCs) [42]. Although MSCs are distinct from Tregs, they are known to induce peripheral tolerance through soluble factors such as TGF-β1, which plays a central role in the induction and maintenance of Foxp3, as well as PGE2, IDO, and IL-10, and through cell–cell contact [110]. The systemic administration of MSCs increases endogenous Tregs and suppresses Th17 cells [42,111]. In other words, MSCs can be considered another therapeutic option that achieves the same effect through a mechanism distinct from that of direct Treg infusion. However, Kikuiri et al. did not identify primary mediators in MSCs [42].

Thus, through bidirectional interventions, the removal and replenishment of Tregs have been shown to be defining factors in this condition [42]. In other words, MRONJ is a condition in which a reduction in the number of Tregs determines its onset. However, this conclusion rests on the presence of a clear trigger: the direct reduction in Tregs by the causative drugs. The suppressive activity itself has not been measured. Therefore, the key to treating MRONJ is to replenish and restore the reduced Treg count.

Although the mechanisms and therapeutic effects discussed in this section are based on animal models, the core findings, namely the reduction in Tregs and increase in IL-17 caused by bisphosphonates, as well as the healing and recovery of Tregs following MSC administration, have been reproduced not only in mice [42] but also in minipigs [109], demonstrating cross-species reproducibility. In humans, cell therapy using the autologous adipose tissue-derived stromal vascular fraction (AT-SVF), which includes MSCs, has been reported to promote soft-tissue and bone healing in MRONJ lesions [112]. However, as this was a prospective study of nine cases without a control group, and immunological markers, including Tregs, were not measured, it remains unclear whether this healing resulted from a Treg-mediated mechanism. Furthermore, because all this evidence and the proposed mechanisms are based on bisphosphonate-related osteonecrosis of the jaw (BRONJ), it remains unclear whether the same mechanism applies to MRONJ caused by other drugs, such as denosumab, which do not act on the mevalonate pathway. Moreover, the pathogenesis of MRONJ is multifactorial [105,113], and only part of the Treg/Th17 axis is discussed in this section.

Therapies aimed at restoring Tregs are currently being investigated for practical applications. In a BRONJ model using minipigs, administration of allogeneic bone marrow-derived mesenchymal stem cells was effective [109], suggesting the potential for “off-the-shelf” cell therapy without requiring patient collection. Cell-free approaches using MSC-derived small extracellular vesicles (sEVs) in place of MSCs are also advancing, and Eid3 contained in MSC-sEVs destabilizes RORγt and thereby suppresses Th17 cells [114]. However, this was demonstrated in a model of experimental autoimmune encephalomyelitis (EAE), and its application in MRONJ requires further verification.

Across the six diseases reviewed above, the state of the Treg suppressive function and the direction in which intervention is required are summarized in Figure 2.

## 4. Cross-Disease Synthesis

In oral diseases, regulatory T cells have most often been discussed from a numerical standpoint: a greater number of Tregs suppresses immunity and is protective, whereas a smaller number permits inflammation and is pathogenic. However, as observed across the six diseases in this review, the number of Tregs alone cannot account for the pathology of each disease. It is necessary to examine the suppressive function itself, but studies in which this function has been measured are limited. Table 1 summarizes, for each of the six diseases, whether the suppressive function itself was assayed and what each cited study measured.

Suppressive function has been measured in four diseases: oral lichen planus, OSCC, periodontitis, and SjD. Of these, oral lichen planus and OSCC showed opposite results in the same assay. When the suppressive function of peripheral blood Tregs was measured, Tregs in oral lichen planus were lower than in healthy controls [6], whereas Tregs in OSCC were higher [92]. Both studies compared the suppressive capacity of peripheral blood CD4^+^CD25^+^ T cells from patients with that of healthy controls. The responder cells and the cell ratios differ from study to study, however, and every suppression assay in humans rests on fewer than ten patients [6,47,48,92]; a direct quantitative comparison is therefore limited. In humans, FOXP3 is an intracellular transcription factor, so FOXP3^+^ cells cannot be sorted live; suppression assays are therefore performed on cells sorted by other markers, such as CD4^+^CD25^+^ or CD4^+^CD25^high^ [47]. In periodontitis, the anti-osteoclastogenic capacity of sorted Tregs was reduced in a mouse experimental system [7]. In SjD, the suppressive function has been reported to be comparable to that of controls [47,48], whereas the responsiveness of STAT5 to IL-2 stimulation was reduced [57], so that the conclusion differs depending on the index used. In mice, co-transfer of equal numbers of Tregs showed that wild-type Tregs suppressed lymphocytic infiltration of the lacrimal gland and IFN-γ production and the loss of tear production, whereas Stim1/2-deficient Tregs did not, despite being present in the same numbers [44].

What recurs across diseases at the level of measured indices is the destabilization of Foxp3 and the acquisition by Tregs of Th17 characteristics they should not possess. In periodontitis and peri-implantitis, Foxp3 is destabilized, and cells on the suppressive side acquire Th17 characteristics, such as IL-17 production; this plasticity is common to both conditions. A similar tendency has been reported in the peripheral blood of oral lichen planus, but this has not been confirmed in lesional tissue [6,63], indicating a difference between the compartments. Specifically, transient IL-17A production by Foxp3 fate-tracked cells has been reported in periodontitis [86], and simultaneous upregulation of FOXP3 and RORγt in peri-implantitis [85]. What has been shown in peri-implantitis extends only to an increase in FOXP3^+^ cells and a change in the composition of suppression-related molecules; the suppressive function itself has not been measured [85,87]. However, the driving mechanisms differ between the diseases. In oral lichen planus, no study has demonstrated this mechanism in the disease itself. Therefore, FOXP3 destabilization in this disease remains no more than an inference drawn from general knowledge, including findings in other diseases. In periodontitis, TSDR methylation showed no significant change, leaving the reduction in Foxp3 unexplained [7], whereas in peri-implantitis, it appears as a decrease in the stability marker NRP-1 [87]. NRP-1 has not been examined in Tregs in other oral diseases, and cross-disease comparisons using this index remain a task for future research. In SjD, moreover, the basis of the dysfunction shown in mice is different, being attributed to impaired Treg-specific calcium signaling (store-operated Ca^2+^ entry via STIM1/2), and its downstream response is skewed toward Th1/IFN-γ rather than Th17 [44]. What emerges as common is that the single point at which cell counts are taken does not represent their function; the molecular abnormality that produces this, and the direction of inflammation, differ by disease. For OSCC, metabolic reprogramming of the tumor environment is envisaged as the mechanism supporting the suppressive function. However, this has been generally demonstrated in cancer, and in OSCC itself, the findings remain at the level of association. Periodontitis and peri-implantitis are both characterized by biofilm-driven bone resorption; however, peri-implantitis is also associated with specific conditions, including foreign-body reactions and a poorly vascularized environment.

This is complemented by three further considerations. First, localization must be considered alongside function. The action of Tregs depends not only on their function but also on whether sufficient numbers reach the site where they are needed. In periodontitis, locally produced CCL22 recruits CCR4^+^ Tregs to lesions, thereby limiting bone resorption [77,78]. However, this recruitment approach has not yet been tested in peri-implantitis. Second, the significance of the function depends on the context. In OSCC, a high number of FOXP3^+^ cells is associated with poor prognosis [100], whereas in head and neck squamous cell carcinoma, there are reports of an association with favorable prognosis [101,102]; the heterogeneity of FOXP3^+^ cells, among other factors, can change whether Tregs are beneficial or harmful. Therefore, uniformly lowering or raising function is not always optimal. Third, there are limitations in the basis of comparison. Studies on different diseases have identified Tregs using various markers and methods (FOXP3 immunostaining, CD4^+^CD25^+^, flow cytometry for CD25-high and CD127-low cells, transcription-factor mRNA, and others), and this variation in indices makes rigorous cross-disease comparison of Treg status difficult.

MRONJ occupies a different position among these six diseases. In MRONJ, Treg numbers are reduced; however, the suppressive function of Tregs has not yet been measured. Depletion of Tregs with an anti-CD25 antibody worsens the lesion, and Treg transfer restores healing [42], establishing that Tregs govern this condition. Causative drugs reduce Tregs and simultaneously activate Th17 cells, thereby disrupting the Th17/Treg balance and immune homeostasis. Accordingly, the therapeutic approach is not to restore Treg function but to replenish and increase Treg numbers [42,109]. In MRONJ and periodontitis, the protective role of Tregs towards the bone is shared, but the mechanisms are the opposite. In an experimental model of periodontitis, Tregs sorted from healthy mice suppressed osteoclast formation [7]. In MRONJ, osteoclasts suppressed by zoledronate recover upon Treg transfer [42]. The points of action differ between the two conditions. Zaiss et al. demonstrated direct suppression of precursor cells, whereas the return of osteoclasts in MRONJ is thought to result from the correction of the inflammatory environment and normalization of the healing process [115]. Similarly, the weight of the evidence was not symmetrical. On the side where Tregs suppress osteoclast formation, there is quantification from co-culture [7,115], whereas in MRONJ, the evidence is a staining finding in immunocompromised mice, for which no quantitative assessment has been made [42].

In terms of function, the handling of Tregs in each disease takes a different form from the conventional view argued by numbers [5]. In OSCC, where the suppressive function is enhanced, weakening Treg-mediated suppression by targeting metabolic pathways is a promising approach to enhance the effect of immunotherapy. In diseases with persistent inflammation, the converse applies: restoring and maintaining suppression through low-dose IL-2 [116], stabilization of FOXP3, or the recruitment of functional Tregs to the site. In MRONJ, in which Tregs are numerically reduced, replenishment with MSCs or Tregs is central to treatment. Interventions in opposite directions are required for different disease states. Therefore, it may be useful to identify factors that can regulate Treg function in both directions. GDF15, for example, stabilizes FOXP3 by suppressing STUB1, thereby enhancing the suppressive function of Tregs [98]. This factor could serve as a target for weakening in OSCC, where the suppressive function is enhanced, and conversely, as a means of strengthening immune tolerance in diseases with persistent inflammation. In SjD, serum GDF15 is associated with disease activity, and the authors postulated that it may enhance the function of suppressive cell populations, including Tregs [117]; however, Tregs themselves were not measured.

**Table 1:** Studies of Tregs in six oral diseases: whether the suppressive function itself was assayed, and what was measured in each study.

Disease	Suppressive Function Assayed	Result	Sample	Readout	Reference
Sjögren’s disease (SjD)	Yes	Comparable to controls	Human peripheral blood; MACS-purified CD4^+^CD25^+^ T cells (5 SjD, 5 controls in the assay; 52 SjD and 49 controls in the cohort)	Suppression of autologous CD4^+^CD25^−^ T cell proliferation, 1:1 co-culture	Li X. et al. 2007 [48]
	Yes	Comparable to controls	Human peripheral blood; FACS-sorted CD4^+^CD25^hi^ cells (39 SjD, 30 controls; assay run with cells from 4 patients)	Suppression of ^3^H-thymidine incorporation by CD4^+^CD25^−^ responder cells from controls	Liu C. et al. 2017 [47]
	Yes	Reduced	Mouse; WT or Stim1/2-deficient Tregs co-transferred 1:1 with autoreactive CD4^+^ T cells into *Rag1*^−/−^ hosts (4 mice per group)	Lacrimal gland infiltration, CD4^+^IFN-γ^+^ fraction and tear production	Wang Y.-H. et al. 2024 [44]
	No	—	Human peripheral blood; Tregs (functional subset of 51, from 97 SjD and 50 controls)	Frequency of pSTAT5^+^ Tregs after IL-2 stimulation (phospho-flow)	Keindl M. et al. 2022 [57]
Oral lichen planus (OLP)	Yes	Reduced	Human peripheral blood; CD4^+^CD25^+^ T cells (3 reticular OLP, 3 matched controls; 20 reticular and 20 erosive OLP in the cohort)	Suppression of CFSE-labeled autologous CD4^+^CD25^−^ T cell proliferation (significant at the 1:0.5 ratio)	Zhou L. et al. 2016 [6]
	No	—	Human OLP lesions (8 normal mucosa, 7 reticular, 8 atrophic, 4 ulcerative)	FOXP3^+^ cell counts; proportion of FOXP3^+^CD15s^+^ cells (in situ staining)	Schreurs O. et al. 2016 [63]
Periodontitis	Yes	Reduced	Mouse ligature-induced periodontitis; CD4^+^CD25^+^Foxp3^+^ cells sorted from cervical lymph nodes	Suppression of TRAP^+^ osteoclast formation in co-culture with bone marrow macrophages	Alvarez C. et al. 2020 [7]
	No	—	Mouse infected with *Aggregatibacter actinomycetemcomitans*	Alveolar bone loss and inflammatory cell influx after Treg inhibition with anti-GITR antibody	Garlet G.P. et al. 2010 [73]
	No	—	Human periodontitis tissue (10 stage IV grade C, 10 stage III grade B, 10 controls)	FOXP3^+^ and CD25^+^ cell counts (immunohistochemistry)	Da Motta R.J.G. et al. 2020 [74]
Peri-implantitis	No	—	Human peri-implant tissue (15 healthy, 20 peri-implantitis)	FOXP3 and RORγt mRNA (RT-qPCR); preliminary report	Giro G. et al. 2021 [85]
	No	—	Human granulation tissue (23 peri-implantitis, 23 explanted, 20 healthy)	FOXP3, HELIOS, NRP1, TGFB1, IL35B mRNA; FOXP3^+^ cells and NRP-1^+^ area; IL-10, TGF-β1, IL-35 protein	Cafferata E.A. et al. 2025 [87]
Oral squamous cell carcinoma (OSCC)	Yes	Enhanced	Human peripheral blood; magnetically sorted CD4^+^CD25^+^ T cells (9 OSCC patients, 10 controls)	Suppression of PHA-stimulated allogeneic PBMC from healthy donors (1:10 co-culture)	Gasparoto T.H. et al. 2010 [92]
	No	—	Human peripheral blood; oral cavity subgroup (19 of 112 HNSCC patients) and 31 healthy donors	Frequencies of CD45RA^−^FOXP3^high^ and CD45RA^+^FOXP3^low^ Tregs	Sun W. et al. 2014 [93]
	No	—	Human OSCC tumor tissue (106 patients)	Tumor-infiltrating FOXP3^+^ cell count; high count associated with worse prognosis	Hayashi T. et al. 2021 [100]
	No	—	Human HNSCC tumor tissue (205 FFPE specimens)	Tumor-infiltrating FOXP3^+^ cell count; high count associated with longer recurrence-free and overall survival	Seminerio I. et al. 2019 [101]
	No	—	Systematic review and meta-analysis of published HNSCC cohorts (9 studies reporting FOXP3)	Pooled hazard ratio for overall survival in HPV-negative patients (HR 0.80)	de Ruiter E.J. et al. 2017 [102]
Medication- related osteonecrosis of the jaw (MRONJ)	No	—	Mouse treated with zoledronate and dexamethasone; peripheral blood	Frequency of CD4^+^CD25^+^Foxp3^+^ cells; Tregs/Th17 ratio	Kikuiri T. et al. 2010 [42]
	No	—	Mouse tooth-extraction model of BRONJ	Exposed bone and socket healing after anti-CD25 depletion or systemic Treg transfer	Kikuiri T. et al. 2010 [42]
	No	—	Minipig BRONJ model; peripheral blood and serum	Proportion of Foxp3^+^ Tregs in blood; serum IL-17	Li Y. et al. 2013 [109]

“Suppressive function assayed” is Yes when the study measured the suppressive activity of Tregs itself, and No when it measured cell numbers, phenotype or molecular expression only. “Result” is the outcome of that suppression assay, expressed relative to the control Tregs assayed in the same study, as comparable to controls, reduced or enhanced. It is therefore given only for the rows marked Yes, and is shown as a dash for the rows marked No, in which the suppressive function was not measured; what those studies measured instead is given in the Readout column. Rows within a disease are separated by dotted lines and correspond one-to-one to the cited studies.

## 5. Conclusions

This review re-examines the role of Tregs in oral diseases from the perspective of their immunosuppressive function rather than their abundance. On this basis, this function has been measured in few diseases. The significance of this perspective is not limited to the oral cavity. The oral cavity is a setting in which Treg function can be either impaired or excessive, and the principles obtained here may extend to the control of Tregs in systemic autoimmunity and cancer. Substantial challenges remain. In peri-implantitis and MRONJ, suppressive function has not been measured. Moreover, what happens to Tregs in each disease and the molecular basis of these changes remain insufficiently understood. Most studies to date have used Tregs from the peripheral blood or draining lymph nodes, and few studies have directly assessed the function of Tregs at the site of disease onset.

The absence of standardized methods for assessing the suppressive function also makes cross-disease comparisons difficult. Knowledge of factors that regulate Treg function remains insufficient. Factors such as GDF15, which can both enhance and weaken Treg function, need to be examined for their relationship with Tregs at the disease site under oral conditions. Resolving these issues will be key to enabling function-based control of Tregs. Careful consideration of insights gained from future studies focusing on Treg function may provide important therapeutic implications and contribute to the development of new therapeutic concepts.

## Figures and Tables

**Figure 1:**
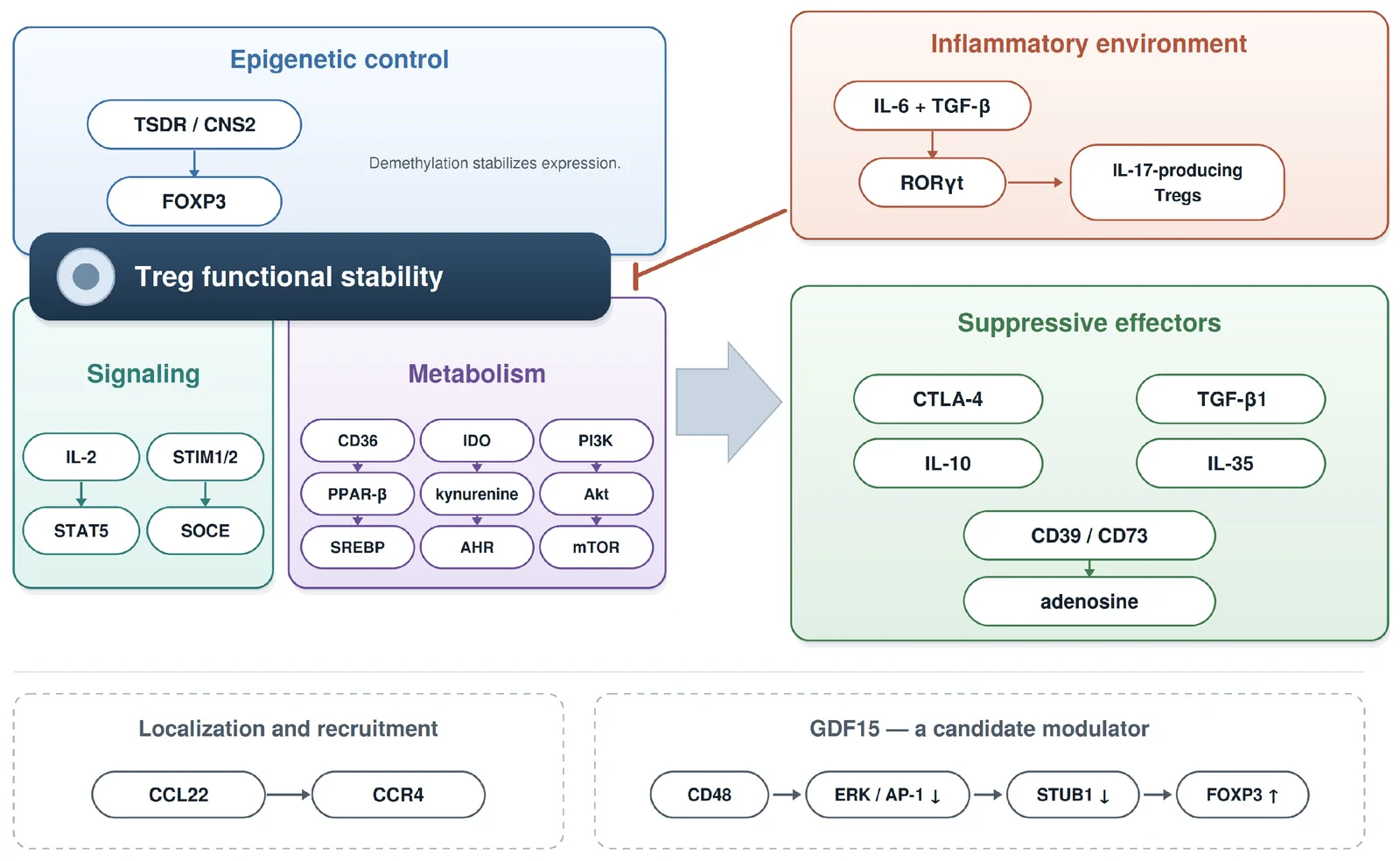
Pathways that govern the functional stability of Tregs. The left-hand side shows the three layers that sustain stability. In epigenetic control, demethylation of the TSDR/CNS2 stabilizes FOXP3 expression, but is not sufficient on its own. Signaling and metabolism are both required to maintain a stable Treg. The inflammatory environment at the top right is an element that undermines stability: IL-6 and TGF-β induce RORγt, drive IL-17 production in FOXP3^+^ cells, and give rise to cells that have lost FOXP3 expression. The bar directed at Treg functional stability in the center indicates that this pathway undermines functional stability. The lower right shows the suppressive effectors, the output of a stable Treg. The dashed boxes at the bottom are shown as axes separate from the suppressive function itself. Localization and recruitment determine whether Tregs reach the lesion. GDF15 is a candidate for shifting Treg function in either direction.

**Figure 2:**
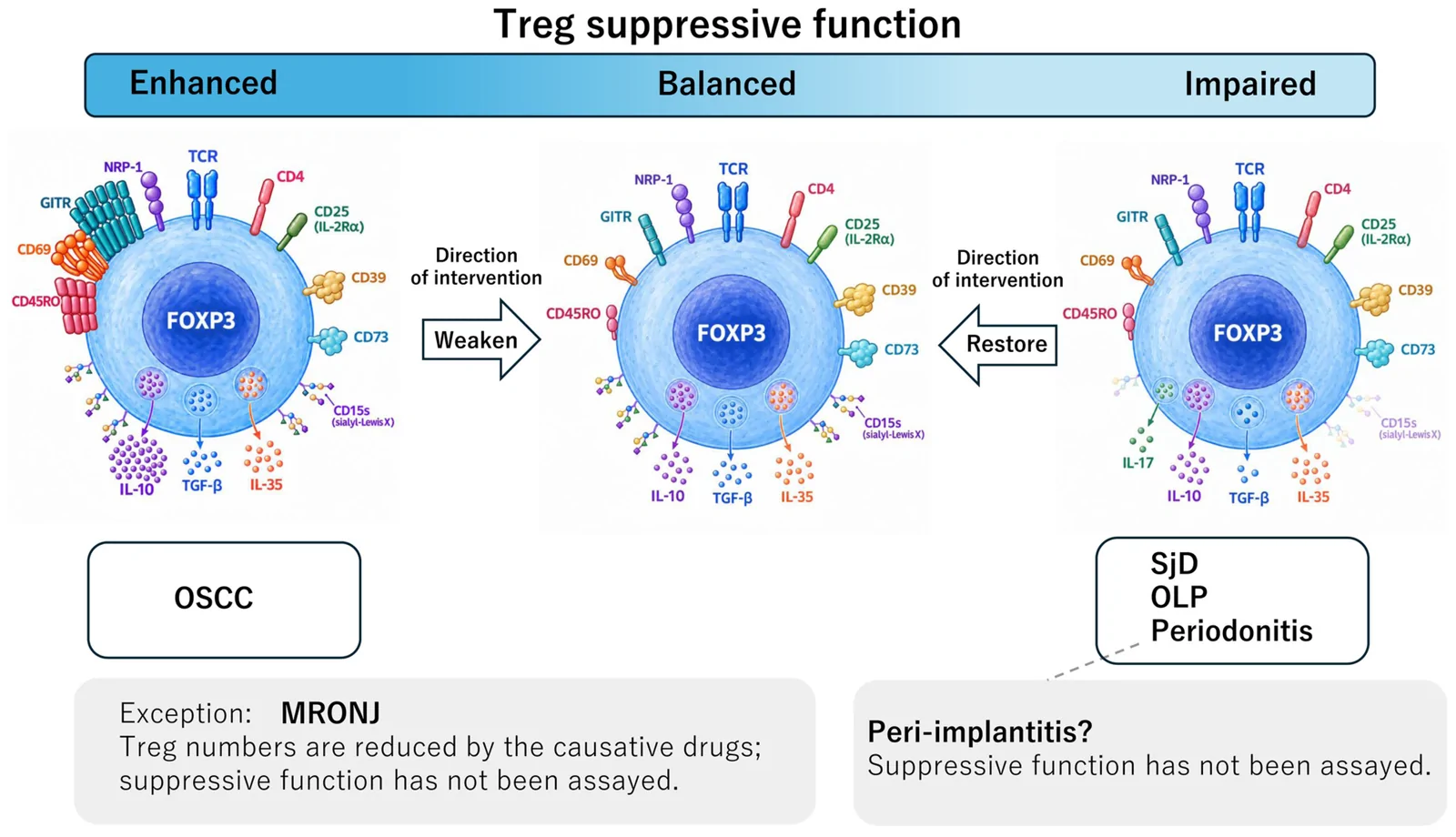
**Direction of intervention required in each oral disease, shown on an axis of Treg suppressive function.** Oral squamous cell carcinoma is placed at the enhanced end of the axis, and Sjögren’s disease, oral lichen planus, and periodontitis at the impaired end. The three cells below the axis represent a Treg in each state. The same identification markers, namely CD4, TCR, CD25, and nuclear FOXP3, together with CD39, CD73, NRP-1, and CD15s, are drawn on all three cells, indicating that the enhanced and impaired states are not distinguished by the markers used to identify Tregs. Only the molecules for which a direction of change has been reported differ between the cells: GITR, CD45RO, CD69, and IL-10 are increased in the enhanced state [92], whereas CD15s [63] and TGF-β [6] are decreased and IL-17 [6,86] appears in the impaired state. The number and intensity of symbols indicate the relative level of expression or secretion. The arrows show the direction in which intervention is required in each state, toward a balanced suppressive function. Peri-implantitis and medication-related osteonecrosis of the jaw are placed outside the axis because the suppressive function has not been assayed in either condition. In medication-related osteonecrosis of the jaw, Treg numbers are reduced by the causative drugs, and the condition is governed by cell number rather than by suppressive function.

## Data Availability

No new data were created or analyzed in this study. Data sharing is not applicable to this article.

## Abbreviations

The following abbreviations are used in this manuscript:

Tregs	Regulatory T cells
Th17	T helper 17
FOXP3	Forkhead box P3
CTLA-4	Cytotoxic T-lymphocyte-associated antigen 4
pTregs	Peripherally induced Tregs
iTregs	Inducible Tregs
tTregs	Thymus-derived Tregs
TGF-β	Transforming growth factor β
TSDR	Treg-specific demethylated region
CNS	Conserved noncoding sequence
RORγt	Retinoic acid receptor-related orphan receptor γt
ACC1	Acetyl-CoA carboxylase 1
STAT5	Signal transducer and activator of transcription 5
STIM	Stromal interaction molecule
CD36	Cluster of differentiation 36
PPAR-β	Peroxisome proliferator-activated receptor β
SREBP	Sterol regulatory element-binding protein
IDO	Indoleamine 2,3-dioxygenase
PI3K	Phosphoinositide 3-kinase
Akt	Protein kinase B
mTOR	Mechanistic target of rapamycin
CCL22	C-C motif chemokine ligand 22
CCR4	C-C chemokine receptor type 4
ERK	Extracellular signal-regulated kinase
AP-1	Activator protein 1
STUB1	STIP1 homology and U-box containing protein 1
SjD	Sjögren’s disease
SOCE	Store-operated Ca^2+^ entry
WT	Wild-type
OLP	Oral lichen planus
ATRA	All-trans retinoic acid
PICF	Peri-implant crevicular fluid
OPG	Osteoprotegerin
NRP-1	Neuropilin-1
FI	Failed implants
SI	Successful implants
NI	No implants
OSCC	Oral squamous cell carcinoma
AHR	Aryl hydrocarbon receptor
GDF15	Growth differentiation factor 15
HPV	Human papillomavirus
PD-1	Programmed cell death protein 1
ICIs	Immune checkpoint inhibitors
MRONJ	Medication-related osteonecrosis of the jaw
BRONJ	Bisphosphonate-related osteonecrosis of the jaw
FPP	Farnesyl diphosphate
GGPP	Geranylgeranyl diphosphate
MSCs	Mesenchymal stem cells
AT-SVF	Adipose tissue-derived stromal vascular fraction
sEVs	Small extracellular vesicles
